# Recent methods for polygenic analysis of genome-wide data implicate an important effect of common variants on cardiovascular disease risk

**DOI:** 10.1186/1471-2350-12-146

**Published:** 2011-10-26

**Authors:** Matthew A Simonson, Amanda G Wills, Matthew C Keller, Matthew B McQueen

**Affiliations:** 1 Department of Psychology, University of Colorado Boulder, USA; 2 Department of Integrative Physiology, University of Colorado Boulder, USA; 3 Institute for Behavioral Genetics, University of Colorado at Boulder, Boulder, CO 80303, USA

## Abstract

**Background:**

Traditional genome-wide association studies are generally limited in their ability explain a large portion of genetic risk for most common diseases. We sought to use both traditional GWAS methods, as well as more recently developed polygenic genome-wide analysis techniques to identify subsets of single-nucleotide polymorphisms (SNPs) that may be involved in risk of cardiovascular disease, as well as estimate the heritability explained by common SNPs.

**Methods:**

Using data from the Framingham SNP Health Association Resource (SHARe), three complimentary methods were applied to examine the genetic factors associated with the Framingham Risk Score, a widely accepted indicator of underlying cardiovascular disease risk. The first method adopted a traditional GWAS approach - independently testing each SNP for association with the Framingham Risk Score. The second two approaches involved polygenic methods with the intention of providing estimates of aggregate genetic risk and heritability.

**Results:**

While no SNPs were independently associated with the Framingham Risk Score based on the results of the traditional GWAS analysis, we were able to identify cardiovascular disease-related SNPs as reported by previous studies. A predictive polygenic analysis was only able to explain approximately 1% of the genetic variance when predicting the 10-year risk of general cardiovascular disease. However, 20% to 30% of the variation in the Framingham Risk Score was explained using a recently developed method that considers the joint effect of all SNPs simultaneously.

**Conclusion:**

The results of this study imply that common SNPs explain a large amount of the variation in the Framingham Risk Score and suggest that future, better-powered genome-wide association studies, possibly informed by knowledge of gene-pathways, will uncover more risk variants that will help to elucidate the genetic architecture of cardiovascular disease.

## Background

Cardiovascular disease (CVD), the pathologies associated with the heart and its vascular structure, are a leading cause of death worldwide [1]. Many environmental risk factors exist for the development of CVD, including smoking, hypertension, diabetes, obesity, as well as personality characteristics such as competitiveness and a Type A behavior pattern[2-4]. Previous studies have also determined that the development of CVD has a strong genetic component, with heritability estimates ranging from 38% to 57% [1,5].

Over the past decade, many large-scale efforts have attempted to find genetic factors associated with CVD outcomes using single nucleotide polymorphism (SNP) markers. Several genome-wide association (GWAS) studies have been successful at identifying genetic variants associated with CVD and related phenotypes [6]. However, the effect sizes attributable to these variants have been small. A sample of previously reported association results and their respective effect size are shown in Table 1. Despite strong statistical evidence, the effect size attributable to any given putative genetic variant is, at best, modest. These results suggest that while GWAS studies have had some success, they currently only explain a very small amount of the genetic risk and heritability for many complex phenotypes, including CVD [7].

**Table 1:** Summary of gene/locus features identified through GWAS in selected previous studies for CVD related phenotypes

CVD related phenotype	Region	Reported gene(s)	Strongest SNP-risk allele from study	Risk allele frequency	P-value	Effect size	Reference
Coronary heart disease	10p11.23	*KIAA1462*	rs3739998-C	0.44	1 × 10-11	1.15*[1.11-1.20]	[42]
Heart Failure	15q22.31	*USP3*	rs10519210	0.03	1 × 10-8	1.53*[1.05-2.24]	[43]
Mortality with heart failure	3p22.2	*CMTM7*	rs12638540-G	0.043	3 × 10-7	1.53*[1.01-2.31]	[44]
Coronary heart disease	3q22.3	*MRAS*	rs9818870-T	0.15	7 × 10-13	1.15 *[1.11-1.19]	[42]
Coronary heart disease	6q25.3	*SLC22A3, LPAL2, LPA*	4-SNP haplotype-2	0.02	4 × 10-15	1.82*[1.57-2.12]	[45]
Coronary heart disease	9p21.3	*Intergenic*	rs1333049-C	0.47	3 × 10-19	1.36* [1.27-1.46]	[46]
Coronary heart disease	9p21.3	*CDKN2A, CDKN2B*	rs1333049-C	0.47	1 × 10-13	1.47* [1.27-1.70]	[7]

The genetic effect refers to the OR and [95% CI]. All listed values are from [6]

The modest number of associated variants found in previous GWAS studies that, together, explain only a small amount of variation in risk likely reflects that a large number of genetic causal variants, each explaining a small amount of risk, contribute to heart disease[5,8,9]. We refer to this as the "polygenic theory" of heart disease risk. Recent applications of polygenic approaches to GWAS data have shown substantial improvement in the ability to predict disease risk from common variants [10-12]. In addition, these approaches have improved our ability to assess the degree to which these common variants contribute to the heritability of disease [10,11]. These methods of polygenic analysis use all SNPs together to examine the net effect of SNPs on disease risk, including loci of small effect that are typically undetectable (given stringent multiple-testing corrections) with traditional association methods [13]. One approach involves generating a genetic risk score that combines the very small effects of all non-significant alleles that are individually insignificant, but when combined have significant predictive ability. Those subjects with higher genetic risk scores generally have higher risk of disease. Another approach examines the pairwise genetic and phenotypic similarity between subjects in a sample, we then examine if those subjects that have greater genetic similarity also have greater phenotypic similarity to each other than is expected by chance. This second approach estimates heritability due to SNPs by using SNPs to generate a genetic similarity matrix between all individuals in the sample. This is analogous to twin and family methods that estimate heritability based on assumed degrees of relatedness. The advantage of this method over the classic twin study approach is that subjects don't have to be siblings and instead come from a sample of unrelated individuals. Another very important advantage of this method is that family and twin studies rely on assumptions about the causes of similarity between family members (e.g., that genes alone explain the greater similarity of monozygotic over dizygotic twins). This method avoids these assumptions due to the fact that subjects do not come from the same family. Furthermore, the polygenic heritability approach provides information on the frequency spectrum of the causal alleles underlying the phenotype--something that twin and family methods cannot do.

Using three different methods of analysis, we show the extent to which the Framingham Risk Score (an important CVD-related phenotype) can be predicted using common genetic variants, as well as how much variation in the phenotype can be explained overall using GWAS data.

## Methods

### GWAS sample

The study sample for this project was derived from the Framingham SNP Health Association Resource (SHARe), (version 6) as available through NCBI's database of Phenotypes and Genotypes (dbGaP). Information on genotypes (Affymetrix 500 K), phenotypes, family structure information, and environmental variables were available in over 9000 participants from three cross-generation enrollment periods.

The Framingham Heart Study (FHS), a project of the National Heart, Lung and Blood Institute (NHLBI), was designed to identify common factors that contribute to risk of cardiovascular disease [14-16]. The FHS has conducted groundbreaking research on CVD outcomes and is cited as the gold standard for cardiovascular genetic epidemiology[17,18]. Details on the design and implementation of the FHS can be found elsewhere [19]. Briefly, 14428 individuals from the town of Framingham, Massachusetts, were recruited over the course of three generations. The majority of FHS subjects underwent extensive physical examinations and lifestyle interviews, enabling the collection of genetic data along with highly detailed phenotypic information [19].

### Phenotype definition and methods

#### Framingham Risk Score Calculation

Calculation of the Framingham Risk Score was based on the methods described by D'Agostino et al. (2008). Briefly, D'Agostino et al. (2008) developed a risk score from participants in the Framingham Heart Study to assess CVD (defined as a coronary death, myocardial infarction, coronary insufficiency, angina, ischemic stroke, hemorrhagic stroke, transient ischemic attack, peripheral artery disease, or heart failure) risk within the next 10 years. In the present study, this same method was used to assign risk scores to the current set of participants to be used as the phenotypic endpoint. These scores were calculated for men and women [separately] using the predictors of: (1) age, (2) HDL, (3) total cholesterol, (4) systolic blood pressure, (5) treatment status of systolic blood pressure, (6) smoking status, and (7) diabetic status. See D'Agostino et al. (2008) for a detailed description of the risk score calculation method [20]. The calculated regression coefficients for risk score are presented in table 2.

**Table 2:** Regression coefficients used in models generating Framingham risk scores for 10-year risk of general cardiovascular disease

Men*			Women*	
**Variable**	**Beta**	**p-value**	**Beta**	**p-value**

Log of Age	3.06117	p < .0001	2.32888	p < .0001
Log of Total Cholesterol	1.1237	p < .0001	1.20904	p < .0001
Log of HDL Cholesterol	-0.93263	p < .0001	-0.70833	p < .0001
Log of SBP if not treated	1.933303	p < .0001	2.76157	p < .0001
Log of SBP if treated	1.99881	p < .0001	2.82263	p < .0001
Smoking	0.65451	p < .0001	0.52873	p < .0001
Diabetes	0.57367	p < .0001	0.69154	p < .0001

The 10-year risk for women can be calculated as 1-0.95012^exp(ΣβX - 26.1931) ^where Beta is the regression coefficient and × is the level for each risk factor; the risk for men is given as 1-0.88936^exp(ΣβX - 23.9802) ^[20]

Complete details on the available data, including assessment protocols can be found in the Framingham SHARe documentation. We note here that blood pressure was measured twice at each examination and the mean of these two measures was used when the participant had both scores. Cholesterol levels were determined by standard enzymatic methods while smoking and hypertensive treatment status was evaluated by participant and physician report. Diabetic status was defined as having definite diabetes (either being treated for diabetes or having a blood sugar reading of greater than or equal to 200 mg/dL).

For the original cohort, age, total cholesterol, blood pressure, smoking and diabetic status were assessed at Exam 1. HDL levels were not available for this cohort until Exam 9, making this measure an unusable predictor for risk score of this cohort. Therefore, a mean HDL level of all remaining participants from Exam 9 was calculated and applied to the original cohort individuals in conjunction with their Exam 1 scores. All predictors in the offspring and 3^rd ^generation cohorts were calculated from their first exam. With the exception of missing HDL levels in the original cohort, risk scores were only calculated for individuals with complete predictor information from the first exam.

### Genotyping methods and quality control

#### Traditional GWAS and Polygenic Prediction

Genome-wide genotypes and detailed clinical data on subjects have been made available to researchers through the SHARe (SNP-Health Association Resource) project. Unfiltered genotype data used in our study contained 9236 individuals genotyped for 500,568 SNPs (from the Affymetrix 500 K mapping array). We used PLINK, the whole genome association analysis toolset, in combination with R statistical computing software to perform quality control procedures [10,21].

Subjects were excluded if genotyping rates were less than 95%. Individuals were also excluded if the predicted sex based on X-chromosome genotypes did not match the recorded sex. Evidence of non-random genotyping batch effects was inferred from identity-by-missingness (IBM) clustering of subjects. A batch covariate was generated for each subject and used to control for any batch effects. Subjects who were outliers with respect to estimated heterozygosity, those greater than 3 standard deviations from the mean, were excluded. All close relatives of individual subjects, based on mean identity-by-descent (IBD; PIHAT in PLINK) values indicating relatedness of less than 2^nd ^degree relatives, were excluded from the sample. All subjects with an identity-by-state (IBS) genetic distance from the sample mean of more than 3 standard deviations were considered outliers with respect to genetic ancestry and were pruned from the sample. This was also confirmed through visual inspection of Multidimensional scaling (MDS) plots. Markers were excluded if (1) genotyping rates were less than 95%, (2) minor allele frequencies were less than 0.01, and (3) if p-values from the Hardy-Weinberg Equilibrium (HWE) test were less than 1 × 10^-4^. Evidence of non-random genotyping failure was examined, as inferred by flanking haplotypic background and significant markers were excluded (PLINK mishap test, p < 1 × 10^-6^). We also removed individuals who had missing values for any covariates or phenotypic data. After quality controls, the remaining sample consisted of 1772 individuals genotyped on 258891 SNPs.

#### Polygenic Heritability Analysis

To minimize the chance that our polygenic heritability findings could be due to confounders (e.g., SNP batch effects) we used additional, very stringent quality control procedures for this analysis, as suggested in [11]. Only those quality control methods that differ from those detailed above are listed below; otherwise all previously mentioned quality control procedures and generation of covariates were the same.

Markers were excluded if p-values from HWE tests were less than .05. Evidence of non-random genotyping failure was examined, as inferred by flanking haplotypic background and significant markers were excluded (PLINK mishap test, p < 1 × 10^-10^)[11]. Subjects were excluded if genotyping rates were less than 99%. All close relatives of individual subjects, based on pairwise IBD (PIHAT) values greater than .025, were excluded from the sample when the GCTA software package was used to estimate heritability. We also removed individuals who had missing values for any covariates or phenotypic data. After quality controls, the remaining sample consisted of 1270 individuals genotyped on 250378 autosomal SNPs.

The polygenic heritability method involves first calculating the pairwise relatedness from SNPs between all individuals in a sample. When using the GCTA software package, any subjects that were related to any other subject with a degree greater than .025 IBD were dropped from the analysis altogether. This resulted in a significant loss of sample size (from 6725 to 1270) and thus a large reduction in power and an increase in standard error of the heritability estimate. To circumvent this large drop in sample size, we utilized an additional method of estimating heritability from pairwise relatedness based on a Haseman-Elston (H-E) regression framework [21]. In a traditional H-E regression analysis, genetic relatedness is based on knowledge of pedigree and is regressed on trait difference between twins (either MZ or DZ). This generates an estimate for how much genetic similarity predicts phenotypic similarity. Our method uses a conceptually similar approach, but instead of using pedigree information, SNPs are used to determine the degree of genetic similarity between unrelated individuals. Using H-E regression, only those individual pairwise comparisons > .025 IBD (rather than entire subjects) were removed. This allows for the genetic relatedness information from all comparisons between subjects (other than those between close relatives) to be utilized in the analysis. Due to the non-independence between pairwise comparisons (the pairwise relationships from the same person are not independent of one another), we used bootstrapping to determine all significance levels and confidence intervals for H-E regression results. After quality controls, the sample used in the H-E regression analysis consisted of 6725 individuals genotyped on 250378 autosomal SNPs.

### Statistical analysis methods

We performed three methods of genome-wide analysis using population-based methods. For all analyses, a logarithmic transform of the cardiovascular disease phenotype score ensured that outliers in the data did not have unusual leverage on the estimates of our analyses, as well as preventing the assumption of normally distributed errors from being violated [22]. Analysis was also performed on untransformed data and effectively resulted in all of the same conclusions presented.

#### Traditional GWAS

Initially a traditional GWAS analysis was performed on all SNPs using Framingham risk score as the target phenotype. Using the PLINK software package (v1.07) with the linear models option, a multiple linear regression test was performed on all quality controlled SNP data using 1772 individuals [10]. An additive mode of inheritance was assumed and empirical p-values were generated for association with the quantitative phenotype at each locus after controlling for age, sex, genotyping batch effects, and (to control for the effects of population stratification) the 20 most significant principal components. A Manhattan plot and a Quantile-Quantile (Q-Q) plot were used to visualize association results. Prior to the analysis, we adopted the genome-wide significance threshold of p < 5 × 10^-8 ^to account for multiple testing [23]. SNP call intensities were examined to determine whether significant SNPs were aberrations caused by poor quality genotyping. Significant SNPs within single genes based on NCBI Build 36.3 were then examined in *Entrez *Gene and *PubMed *to determine if any of these genes had previous associations with phenotypes thought to be related to cardiovascular disease.

#### Polygenic Prediction

Our second approach used a predictive polygenic model that effectively estimates the ability of common variants to predict the phenotypic score. Polygenic inheritance is expected to apply to many complex traits, CVD not withstanding [24]. Thousands of small effects that are indistinguishable from background noise when performing a traditional GWAS can collectively account for a large proportion of the risk variation. Given the available sample size, we used a 10 fold cross-validation technique to minimize any effects of sampling error on polygenic prediction estimates [25]. Methods of cross-validation are commonly used when the intent is to estimate how accurately a prediction model will perform with minimal bias due to the sampled data [26]. This method involved partitioning the data from subjects into 10 discovery and test sets respectively. Each discovery set consisted of 90% of the individuals (N = 1575) from the total data set, while each test set consisted of the remaining 10% of subjects. Each of the 10 test sets was composed of a different 10% of the sample, thus no overlap between any of the test sets existed. For each discovery set, a GWAS analysis was performed [27]. Using the results from this discovery phase, subsets of SNPs composed of those with p-values in the ranges of > .9-1, > .8-.9, > .7-.8, > .6-.7, > .5-.6, > .4-.5, > .3-.4, > .2-.3, > .1-.2, > 0-.1 were generated [13]. The rationale behind grouping subsets of SNPs in ascending 0-.1 p-value bins is to verify the assumptions of a model where many SNPs of small effect are major contributors to CVD risk. With the current sample size, power to detect variants of small effects is individually very low, and by relaxing significance thresholds, power to detect common variants with small effect sizes increases (with a concomitant rise in number of false associations). SNPs from the discovery set in the lower p-value bins should on average have more predictive ability than those in the higher p-value bins, due to the higher ratio of true associations to type I errors [27].

Using the corresponding independent test set (the remaining 10%, N = 175), a genotypic risk score profile was assigned to each individual that represents the net predictive effect of all reference alleles possessed by a given individual. These genotypic risk scores were calculated using PLINK's SNP scoring routine. Each individual's score was calculated by summing the number of reference alleles at each locus multiplied by the value of the Beta for that respective SNP, which were generated from the initial survey of the discovery set [10,13,27]. Linear regression was then used to assess the relationship between risk of cardiovascular disease and the constructed polygenic risk scores in the test group [26]. This same procedure was iterated for each of the 10 sample hold outs, allowing an unbiased estimate (in terms of r^2^) of the ability of common SNPs to predict the Framingham Risk Score.

#### Polygenic Heritability Analysis

Our third approach used two methods (restricted maximum likelihood and H-E regression) to estimate the proportion of the variance in the Framingham Risk Score that is explained by the common SNPs from the FHS data set. Instead of trying to predict heart disease risk from genotype, we examined if similarity in phenotype was significantly predicted by similarity of genotype. We examined if those subjects whose genomes are more similar than is expected by chance also have phenotypes that are more similar than is expected by chance. Using the GCTA software package [28], a genetic relationship matrix was generated based on shared combinations of SNPs and weighted by allele frequency. Then a linear model was fit to these genetic relationships using either a) a restricted maximum likelihood (REML) estimation maximization algorithm, as implemented in GCTA, or b) a linear regression model based on a H-E regression framework to estimate the variance explained by common SNPs [28].

## Results and Discussion

### Traditional GWAS

No individual SNPs were associated with Framingham Risk Score at a genome-wide level of significance after controlling for all covariates. The ten independent SNPs that achieved the lowest p-values (above the significance threshold) are shown in table 3. We further investigated these most significant SNPs to look for any evidence of replication of previous findings. Four of the top ten most significant SNPs were in regions directly associated with CVD or related phenotypes based on previous research. These SNPs are highlighted in table 3.

**Table 3:** Ten most significant SNPs associated with Framingham Risk Score using GWAS

SNP Rank	SNP ID	Region	Gene	Beta	P-value
1	rs17584191	5q23.2	*Intergenic; flanking LMNB1*	0.09579	1.866e-05
**2**	**rs17051776**	**13q13**	*NBEA*	**-0.28380**	**2.987e-05**
3	rs483487	3q26.31	*NLGN1*	0.11820	3.239e-05
4	rs215935	6q14-q15	*Intergenic; flanking TBX18*	-0.08192	4.258e-05
**5**	**rs1862523**	**5p14-p13**	*Intergenic; flanking NPR3*	**-0.08753**	**4.610e-05**
**6**	**rs2111202**	**7q3**	*NRCAM*	**0.09009**	**5.237e-05**
**7**	**rs12611756**	**2p12-p11.1**	*CTNNA2*	**0.08251**	**5.875e-05**
8	rs1491609	3p13	*Intergenic; flanking FOXP1*	-0.08143	5.952e-05
9	rs1346949	18q21.33	*Intergenic; flanking PIGN*	-0.08592	6.730e-05
10	rs17205291	1q42	*Intergenic; flanking KIAA1804*	0.10680	6.860e-05

No single SNP was significant at the genome-wide level after multiple testing correction. SNPs that appear to replicate previous findings are highlighted.

The SNP rs17051776, located within the gene *NBEA *has been previously reported to be associated with blood lipid levels [29]. A strong association was found for SNP rs1862523, which is flanking the 5' end of the gene *NPR3. NPR3 *has been previously reported to be associated with ventricular dysfunction, blood pressure in obesity-associated hypertension [30], as well as a family history of hypertension and cardiovascular disease [31]. The final two SNPs - rs2111202 and rs12611756, are in genes that have been previously reported to be associated with smoking, another factor contributing to Framingham Risk Score [32,33].

### Polygenic Prediction

Two of the ten examined SNP sets (binned by p-values) were able to significantly predict the Framingham Risk Score phenotype, with a third set just missing statistical significance. This suggests a polygenic etiology underlying CVD (as measured via the Framingham Risk Score), as well as the existence of a number of relevant risk variants of small effect across the allele frequency spectrum (see Table 4). While the effect of any single SNP could not be discerned from random noise in the initial GWAS, the combined predictive ability of many SNPs based on score sets provides evidence that some of the risk for CVD is due to common SNPs. The ranges of the significantly predictive bins shows the range of p-values where true associations are maximized relative to the number of type I errors (signal to noise maximization). While a weak overall trend of greater predictive ability is observed as the p-value range of the SNP set becomes more stringent, excessive noise is likely to blame for the apparent lack of this effect in some of the more stringent bins (> .3-.4, > .1-.2, > 0-.1). As expected with a sample of this size, the predictive ability of any SNP set is poor, due in large part to the small number of true associations relative to type I errors in the discovery set across allele frequencies. Examination of the Quantile-Quantile (Q-Q) plot from the initial GWAS confirms that few associations diverge from what is expected by chance, explaining both the general uniformity in number of SNPs in each SNP set, as well as the generally weak predictive ability of each set.

**Table 4:** The number of scoring SNPs that fall into each p-value threshold based on the discovery set is shown.

Target GWAS Statistics for Each Scoring SNP Set			
**P-Value Threshold Range**	**Number of SNPs**	**P-Value**	**Adjusted R^2^**

> 0-.1	24843	0.4974	0
> .1-.2	24201	0.8096	0
**> .2-.3**	**24329**	0.00393*	**0.004177**
> .3-.4	23962	0.971	0
**> .4-.5**	**24269**	0.032033*	**0.002056**
**> .5-.6**	**24500**	**0.06449**	**0.001383**
> .6-.7	24429	0.7129	0
> .7-.8	24429	0.23410	0.0002382
> .8-.9	24397	0.444	0
> .9-1	24508	0.7921	0

Significant ranges and their respective p-values are highlighted.

### Polygenic Heritability Analysis

A linear model was fit to the CVD data using a restricted maximum likelihood (REML) estimation maximization algorithm to estimate the variance explained by common SNPs. This was done three times using comparable algorithms for REML iterations within the GCTA software package. These REML algorithms included 1.) Average Information, 2.) Fisher-scoring, and 3.) Estimation Maximization; all three resulted in approximately the same genetic variance being explained, with heritability estimates between 31% and 34%, as is shown in Table 5. However, none of these three estimates were significantly different from zero due to the large standard errors around the estimates. As explained above, the REML analysis used data from only 1270 subjects, limiting the power of this approach.

**Table 5:** Results of Linear Model analysis using 4 algorithms to estimate heritability V(g)/Vp

	AI Algorithm		Fisher Algorithm		EM Algorithm		HE Regression Algorithm		
**Source**	**Variance**	**SE**	**Variance**	**SE**	**Variance**	**SE**	**Variance**	**SE**	**P-value**

V(g)	0.000668	0.000569	0.000668	0.000551	0.000727	0.000552	0.250341	0.133737	
Vp	0.002151	0.000087	0.002151	0.000086	0.002152	0.000087	1.354157	0.015038	
V(g)/Vp	0.310483	0.263126	0.310483	0.254964	0.337865	0.25492	0.184869	0.098453	0.026*
logL	3222.103		3222.103		3222.098				
n	1270		1270		1270		6725		

Algorithm type and their respective heritability estimates and standard errors are highlighted.

To utilize more of the data and improve power to detect heritability, we also used a linear regression model based on the H-E regression framework to estimate the variance explained by common SNPs [28]. Heritability due to common SNPs was estimated at 18%. Empirical standard error estimates for genetic variance, phenotypic variance, and p-values were all determined using a bootstrapping re-sampling procedure. Due to the much larger sample size used with the H-E regression algorithm, the estimates provided by this model have much smaller standard error than those provided using the REML approach and give a significant estimate for the heritability of cardiovascular disease due to common SNPs (p = .026) as can be seen in Table 5.

## Conclusions

The aim of this study was to use recently developed methods of polygenic analysis in conjunction with traditional GWAS methods to identify SNPs that may be involved in the risk of CVD, to understand how well CVD can be predicted from using all SNPs, and to estimate the variance explained in CVD risk when considering all SNPs together. Using data from the FHS, we performed complimentary methods of analysis examining the genetic factors contributing to Framingham Risk Score, a widely accepted indicator of CVD risk. The use of H-E regression in order to maximize sample size in a sample of related individuals and subsequent bootstrapping to estimate heritability and standard errors represents a novel advance that can be utilized in other samples where individuals are highly related [25,26,28].

Due to linkage disequilibrium, genotyped SNPs tag other, unobserved, variants in the genome. Some have argued that when certain SNP appears to be associated with a disease, it is possible that the SNP itself is not the causal variant [34]. The hypothesis that synthetic association, the process described in the previous sentence, represents a significant portion of the common variant associations observed in the literature today, has been shown to be a very unusual occurance [35]. Given synthetic associations are not the driving force behind the majority of disease associated SNPs in the genome, the polygenic methods employed in this paper, used for both estimation of risk and heritability, provides the first evidence that a substantial proportion of the heritability underlying CVD is due to common causal variants. The results of this study show that SNPs predict a small but statistically significant amount of the genetic risk for CVD (as measured via the Framingham Risk Score).

Although our results provide compelling evidence for the polygenic structure of the genetic architecture underlying CVD, it does not pinpoint where these risk variants reside within the human genome. Nevertheless, the top SNPs identified in the initial GWAS performed, and the genes in which they reside, merits further investigation. One way to further inform predictive polygenic analysis would be to include SNPs that lie within genes and pathways when grouping SNPs into predictive SNP sets. Gene and pathway-based prediction would examine the predictive utility of a group of genetic variants that all belong to the same biological pathway [36]. This method of predictive analysis could help to give a more holistic perspective of the underlying structure of CVD and evaluate the contribution of different biological processes and systems to disease risk [37]. Many SNPs that appear to be insignificant when examined independently, or uninformed sets that are weakly predictive of disease state, could be highly significant and predictive when examined as a set composed of SNPs from genes that work in concert within a pathway [38]. An example of the success of pathway based analysis when applied to GWAS is shown by a recent article where the underlying pathways of 11 diseases are examined and strong findings and replication are observed [39].

Given the small effect sizes of common genetic variants, GWAS studies will have to assemble larger sample sizes in order to identify true genetic association signals arising for individual SNPs. For example, Yang et al (2010)[11] conducted a GWAS involving approximately 180,000 subjects where they were able to explain approximately 10% of the genetic variance in height while simultaneously identifying the significant loci that contribute to this estimate. As sample sizes increase, the power to detect small effects increases. Although the detected polymorphisms may explain little variation individually, with large sample sizes, their cumulative effect should increase, and through pathway analysis and consideration of gene families, such studies can provide important insight into the genetic architecture underlying traits. Similar conclusions have been drawn from recent studies involving the polygenic structure of height as well as schizophrenia [10,11].

One of the weaknesses of the present study was the modest sample sizes (see table 5) used for the GWAS, the polygenic risk score analysis, and the estimation of heritability using the maximum likelihood approach. As explained above, larger sample sizes than the one currently used are needed to detect small effects sizes likely to underlie the genetic variation in CVD. Similarly, it is likely that the ability to predict CVD using SNPs will improve measurably with larger sample sizes, as recently shown to be the case in schizophrenia [10]. The ability to predict a significant portion of disease risk from genotype can result in early intervention and improved treatment. Identification of a molecular profile could be a useful tool for prevention and management of CVD and related disorders [40].

CVD is a complex, highly polygenic disease that is one of the leading causes of death worldwide. This study has demonstrated novel approaches to investigate heritability due to SNPs in samples where individuals are unrelated and has also shown that SNPs can be used to predict CVD. We also provide evidence strongly consistent with the polygenic theory of CVD and show that much of the genetic variation underlying CVD is likely to be due to common causal polymorphisms. Based on these results, we conclude that there remains much information in, and much to be learned by the continued use of SNP panels in the investigation of cardiovascular phenotypes as well as other human diseases. Through the use of these methods in future studies, a better understanding of disease, and improved clinical outcomes are achievable [41].

## Authors' contributions

MS and AW carried out all statistical analyses and pre-analysis organization of data. MS wrote all custom software used during the analysis and was the primary author of manuscript. MK and MM consulted with MS on appropriate statistical and genetic analysis methods to employ, and were directly involved in organizing the structure and revision of the manuscript. All authors have read and approved the final manuscript.

## Pre-publication history

The pre-publication history for this paper can be accessed here:

http://www.biomedcentral.com/1471-2350/12/146/prepub
